# Finding relevant biomedical datasets: the UC San Diego solution for the bioCADDIE Retrieval Challenge

**DOI:** 10.1093/database/bay017

**Published:** 2018-03-16

**Authors:** Wei Wei, Zhanglong Ji, Yupeng He, Kai Zhang, Yuanchi Ha, Qi Li, Lucila Ohno-Machado

**Affiliations:** 1 University of California, San Diego, 9500 Gilman Drive, MC 0728, La Jolla, CA 92093-0728, USA; 2 Department of Computer Science, Northern Kentucky University, Nunn Drive Highland Heights, KY 41099, USA

## Abstract

The number and diversity of biomedical datasets grew rapidly in the last decade. A large number of datasets are stored in various repositories, with different formats. Existing dataset retrieval systems lack the capability of cross-repository search. As a result, users spend time searching datasets in known repositories, and they typically do not find new repositories. The biomedical and healthcare data discovery index ecosystem (bioCADDIE) team organized a challenge to solicit new indexing and searching strategies for retrieving biomedical datasets across repositories. We describe the work of one team that built a retrieval pipeline and examined its performance. The pipeline used online resources to supplement dataset metadata, automatically generated queries from users’ free-text questions, produced high-quality retrieval results and achieved the highest inferred Normalized Discounted Cumulative Gain among competitors. The results showed that it is a promising solution for cross-database, cross-domain and cross-repository biomedical dataset retrieval.

**Database URL**: https://github.com/w2wei/dataset_retrieval_pipeline

## Introduction

Information retrieval techniques have been applied to biomedical research for decades (1–3). As biomedical research evolves over time, information retrieval is also constantly facing new challenges, including the growing number of available data and emerging new data types, the demand for interoperability between data resources, and the change in users’ search behaviors.

The number of publically available biomedical datasets grew exponentially in the last decade. For example, from 2007 to 2018, the number of gene expression datasets in the Gene Expression Omnibus (GEO) database (https://www.ncbi.nlm.nih.gov/geo/, accessed on 29 January 2018) increased from 131 416 to 2 363 254 (as of 29 January 2018), the number of registered studies in ClinicalTrials.gov (https://clinicaltrials.gov/, accessed on 29 January 2018) increased from 49 241 to 264 450 (as of 29 January 2018), and the number of macromolecular structures in the Protein Data Bank (PDB) database (http://www.rcsb.org/pdb/home/home.do, accessed on 29 January 2018) increased from 47 616 to 137 178 (as of 29 January 2018).

Over the last two decades, there were significant increases in the diversity of available biomedical data types for at least two reasons: (i) new technologies resulted in new types of data, such as ‘next generation’ sequencing (NGS) data (4); and (ii) information technology made biomedical data, such as medical images, easier to access (5, 6).

Biomedical datasets are stored in various data repositories that fulfill different functions. Users may need to query various data repositories to collect all desired information. To formulate effective queries, users need knowledge of the research domain and of retrieval systems; however, users may not be aware of all available repositories to query and this may limit their searches.

Users’ search behaviors evolve over time. Formulating queries is not only done by professional librarians, as users want to be self-sufficient (7). In an NIH-wide survey (8), 95% of the respondents agreed that the most common way they obtained information was through independent search, i.e. without external assistance.

The challenge of finding datasets across repositories without specialized assistance needs specialized solutions. The growing amount of heterogeneous data makes it impossible to know for sure where some data of interest will be, and requires effective systems for identifying and ranking relevant datasets; new data types need compatible representation models; interoperability requirements and the change in users’ behaviors require intelligent systems for formulating queries. Much effort has been made to develop such effective and robust systems (9–11). However, existing systems are still focused on just one or a relatively small number of repositories. The biomedical and healthcare data discovery index ecosystem (bioCADDIE) (https://biocaddie.org/, accessed on 29 January 2018) project, an international effort to promote biomedical data discovery, aims at encouraging data sharing, promoting metadata standardization and indexing, and advancing data discovery (12). Specifically, bioCADDIE has developed DataMed (12), a biomedical dataset search engine to help users search across repositories.

DataMed has indexed 2 336 403 datasets from 74 repositories (as of 29 January 2018), and these numbers keep increasing. However, the system faces important challenges, including how to represent datasets in a compact, yet comprehensive fashion, how to effectively formulate queries and how best to rank retrieved datasets. First, existing dataset metadata do not always provide sufficient descriptions of the datasets. Since the metadata from different repositories have been harmonized into the Data Article Tag Suite (DATS) (13) model, detailed information that is specific to a particular data type or repository may not be easily transformed into the DATS format. Second, DataMed is expected to take users’ free-text questions as inputs and reformulate them to comply with the retrieval system. Finally, identifying and appropriately ranking relevant datasets depend on the specific questions a user is trying to answer. Since many users already know some datasets, they are expecting them to appear on top of retrieved lists, which is not always the case. To improve DataMed and overcome some of the abovementioned obstacles, bioCADDIE launched a broad call for the community to participate in the 2016 bioCADDIE Dataset Retrieval Challenge (14) (here on referred to as the ‘Challenge’) to solicit innovative ideas. The Challenge, developed by a team from the University of Texas, is described in an article by Roberts et al. (14). The University of California, San Diego (UC San Diego) team developed a pipeline and examined its performance on the Challenge-provided platform. The pipeline consisted of five main modules:

1. Automatic collection of additional information beyond metadata for existing datasets,
2. Dataset indexing using metadata and the additional information,
3. Query formulation by analysing users’ free-text questions,
4. Dataset retrieval using Elasticsearch (https://www.elastic.co/, accessed on 29 January 2018) and
5. Re-ranking of retrieved results, using multiple algorithms.

### Related work

#### Retrieval systems

Since 2003, the National Center for Biotechnology Information (NCBI) has started building a cross-database search engine, Entrez Global Query Cross-Database Search System (Entrez) (10). In 2012, Entrez provided access to 37 databases that together contain 690 million records (15). This system supports text searching using simple Boolean queries, and it can efficiently retrieve datasets in various formats such as sequences, structures and references. In Europe, the European Bioinformatics Institute (EBI) also created a cross-database search engine, EBI search, to access its biological databases (11). bioCADDIE’s DataMed is different from NCBI and EBI search engines in scope. Additionally, it is open source, allowing the community to propose modifications or leverage the code for their own applications.

##### Dataset representation

Most biomedical dataset retrieval systems are built on the metadata of datasets, rather than on the contents of these datasets. Compared with the content, metadata are more compact and frequently use ontologies to standardize concepts from different sources. Butte and Kohane (16) mapped words in the metadata of GEO datasets to the Unified Medical Language System (UMLS) (17) concepts. Shah et al. (18) developed an ontology-based approach to identify gene expression and protein expression datasets that address the same diseases. They mapped metadata of datasets from a tissue database and a microarray database to ontology concepts, and therefore enabled identification of datasets on specific diseases across these databases.

##### Query formulation

Various methods have been developed to help users formulate effective queries, such as query expansion (19, 20). Dramé et al. (21) explored MeSH thesaurus-based and UMLS-based query expansion methods for information retrieval in the medical domain. Almeida et al. (22) developed a biomedical literature search engine, which included a dedicated module for formulating queries from free-text questions. The module identified key concepts from questions and then expanded them using the UMLS metathesaurus. Abdulla et al. (23) developed an approach to linearly combine different query expansion methods, and significantly improved mean average precision performance.

##### Results ranking

The performance of an information retrieval system is eventually determined by the number of relevant results and the way they are ranked. Re-ranking algorithms can be used to refine the order of retrieved objects. They can be roughly classified into four categories (24): (i) self re-ranking, using the initial results from a search engine to further improve the results in next search; (ii) example-based re-ranking, using query examples to find the desired results; (iii) crowd re-ranking, using online crowdsourcing knowledge; and (iv) interactive re-ranking, involving user interaction to guide the re-ranking process.

## The challenge data and information

The Challenge provided a collection of metadata (The Challenge data: https://biocaddie.org/benchmark-data, accessed on 29 January 2018) from 794 992 biomedical datasets collected from 20 repositories. Due to the diversity of the repositories, datasets varied in contents and formats. For example, a dataset could be a clinical trial documentation from ClinialTrials.org, a comprehensive description of a protein from PDB, or genomic sequences and the associated annotation from GEO. However, all the metadata of the datasets followed the DATS model (13), and the metadata were formatted in both XML and JSON formats.

Users’ questions were formulated in free-text format, such as ‘Search for data of all types that mention ALP gene in an osteosarcoma across all databases’. The questions were artifacts fashioned after TREC topics (TREC topics: http://trec.nist.gov/data/topics_eng, accessed on 29 January 2018) that emulated the tasks to professional librarians. In the Challenge, 51 questions were generated from three use cases (25). Among the questions, six came with judgements (i.e. a list of relevant datasets), 30 example questions came without judgements and 15 test questions that were released in the middle of the Challenge also came with no judgements.

The evaluation followed TREC evaluation procedures for ad hoc retrieval tasks *post hoc* assessment, but without pooling (26). A dataset was judged to be ‘relevant’ if it met all the constraints in the question, or ‘partially relevant’ if it met a subset of the constraints.

Participants could submit up to five automatic or manual runs, although automatic runs were preferred. Judgements were pre-determined but released after the submission deadline.

## Materials and methods

**Figure 1.**
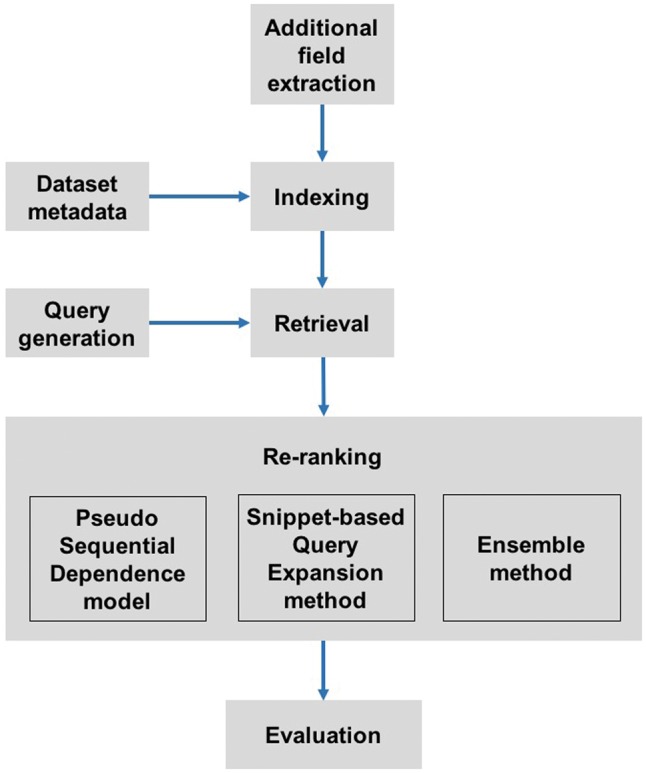
The pipeline for dataset retrieval. Additional information was collected as a supplement to the dataset metadata. Indices were built on the combination of metadata and additional information. Once a query was automatically generated from a user's question, the system retrieved relevant datasets. Next, these datasets were re-ranked using two different algorithms, the pseudo sequential dependence model and the snippet-based query expansion method. The re-ranked results could be further merged to get an averaged result using the Ensemble method. Re-ranked datasets were evaluated on the test set provided by the Challenge organizers.

To achieve real-time retrieval on the extensive collection of datasets, we employed a ‘retrieval plus re-ranking’ strategy to improve the retrieval performance while maintaining efficiency. Our pipeline collected additional information for datasets to supplement the metadata, build indices, automatically analyse free-text questions and generate Boolean queries, retrieve datasets using Elasticsearch, re-rank top datasets from Elasticsearch and evaluate the performance of retrieved results (Figure 1).

### Additional data collection

Retrieval systems depend on comprehensive metadata to obtain user-desired datasets. However, metadata often contain limited information. For example, the metadata of a typical ArrayExpress (https://www.ebi.ac.uk/arrayexpress/, accessed on 29 January 2018) dataset use a ‘description’ field to summarize the study in a few sentences. At the same time, rich information embedded in related sources, such as related publications, may not be fully exploited. To address this challenge, we extended the original metadata of the datasets using online resources in our pipeline. We identified 158 963 datasets that have connections with GEO Series records, and collected the fields ‘Summary’, ‘Title’, and ‘Overall design’ for these datasets from GEO. We named this collection of new fields and values ‘*additional information*’ in our project.

### Indexing

The Challenge provided well-formatted metadata following the DATS model. We developed customized mapping schemas based on the DATS model. In particular, we selected fields in the metadata as ‘standard fields’, which contain the most valuable information about the datasets from each database. The standard fields for each data repository are provided in Supplementary Appendix S1 . The metadata and the additional information were indexed using Elasticsearch. During the construction of indices, fields in the DATS model were classified into three groups: exact matching (e.g. MeSH term), regular string matching (e.g. description) and others (e.g. release date). The text contents of metadata were analysed using the standard tokenizer, English possessive stemmer, lower case filter, non-ASCII character filter, stopword (https://www.ncbi.nlm.nih.gov/books/NBK3827/table/pubmedhelp.T.stopwords/, accessed on 29 January 2018) filter and the Elasticsearch light English stemmer. All MeSH terms and their associated entry terms (i.e. synonyms) were protected against the stemmer.

### Query generation

To enable automatic query generation, we built a module to analyse users’ free-text questions, extract keywords and generate Boolean queries. One example of the free-text question is ‘find data of all types related to TGF-beta signaling pathway across all databases’. In the module, a rule-based filter removed less informative words from questions and kept the key concepts. The less informative words include the English stopwords from Natural Language Toolkit (NLTK) (27) (module detail: nltk.corpus.stopwords.words(‘english’)) and self-defined stopwords: ‘database’, ‘databases’, ‘datasets’, ‘dataset’, ‘data’, ‘related’, ‘relate’, ‘relation’, ‘type’, ‘types’, ‘studies’, ‘study’, ‘search’, ‘find’, ‘across’, ‘mention’, ‘mentions’, ‘mentioning’, ‘i’ and ‘a’.

**Figure 2.**
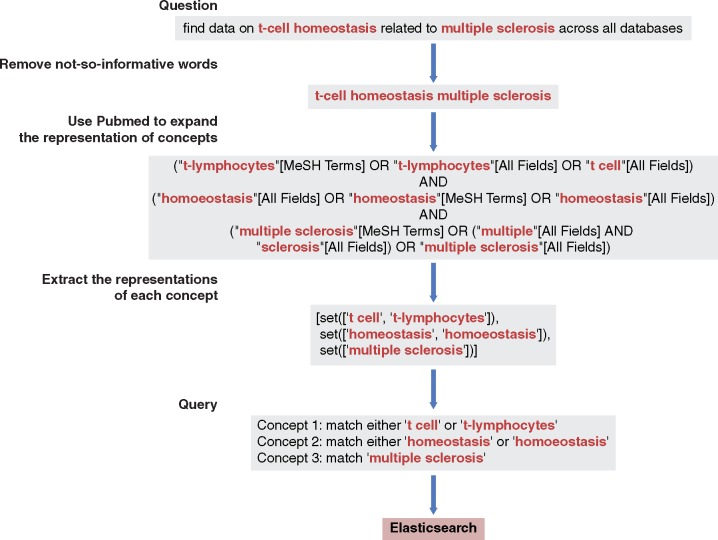
Query interpretation: from a free-text question to a query. Uninformative words were removed using a rule-based method. The query expansion used the same method as PubMed does, relying on NCBI E-utilities.

Next, the remaining words (in our example, ‘TGF-beta signaling pathway’) were passed to PubMed for concept expansion using NCBI E-utilities (28). In this step, key concepts were identified and then expanded. In the example, two concepts, ‘TGF-beta’ and ‘signaling pathway’, were identified in the above question. Then, the ‘TGF beta’ was expanded to two representations, ‘TGFbeta’ and ‘transforming growth factor beta’, while the concept ‘signaling pathway’ was expanded to ‘signal transduction’ and ‘signaling pathway’. Queries generated based on expanded concepts enabled Elasticsearch to search all fields and to retrieve relevant datasets that would be likely missed by the search based on queries without expansion. See Figure 2 for an illustrative example.

Finally, the key concepts and the expanded associations were formulated into nested Boolean queries based on their relationships. Specifically, the representations of the same concept were connected by the ‘OR’ operator, while the different concepts were also linked by the ‘OR’ operator if they satisfied a minimum-match parameter. A concept was recognized as present if the original concept or the expanded associations were observed in the metadata of a dataset. A dataset was retrieved if at least one concept was present. By changing the minimum-match parameter, we performed the search by first retrieving datasets with all concepts present, then obtaining datasets with one fewer concept matching, and so on. Datasets with more matched concepts were ranked higher. Lastly, we only kept the top 5000 datasets for each query.

### Retrieval and re-ranking

We implemented a two-step ‘retrieval plus re-ranking’ strategy. In step 1, Elasticsearch retrieved datasets from the entire collection. In this step, we maximized recall, i.e. we attempted to capture as many relevant or partially relevant datasets as possible in the top 5000 retrieval results, with less focus on the ranking performance. In step 2, we applied re-ranking algorithms to the top 5000 results and aimed at higher inferred Normalized Discounted Cumulative Gain (infNDCG). We explored multiple re-ranking algorithms, and finally adopted a pseudo-sequential dependence (PSD) model, a snippet-based query expansion method (SQEM) and an ensemble method.

#### Pseudo-sequential dependence model

The PSD model was derived from the sequential dependence (SD) model developed by Metzler et al. (29) and Bendersky et al. (30) The original SD models rank documents by considering the unigrams (i.e. single words), ordered bigrams (i.e. two consecutive words) and unordered bigrams (i.e. two words not necessarily consecutive) in documents. In our scenario, the ‘documents’ refer to the metadata of datasets to be re-ranked. In the experiments, we found that neither ordered bigrams or unordered bigrams provided contributions to the performance improvement. One possible explanation is that most keywords are independent of each other, and meaningful bigrams (and n-grams) were likely too sparse and rarely at the intersection of queries and metadata. For example, ‘chromatin modification’ contains more specific information than ‘chromatin’ and ‘modification’, while ‘flu car’ is as informative as ‘flu’ and ‘car’. Bigrams may help with the former example, but not with the latter. In addition, including bigrams results in higher computational complexity, making real-time retrieval difficult. Therefore, we removed the bigram components from the original formula, and modified the unigram component to make it compatible for dataset retrieval tasks, i.e. making ‘whether a word occurs in the metadata’ more important than ‘how many times a word occurs’.

Provided with a query and a list of candidate datasets from Elasticsearch, PSD scores every candidate dataset and re-ranks them all accordingly. The PSD score is defined in Equations (1) and (2), based on Metzler and Croft’s work (29, 31).

(1) $$P=\sum_{q_{i}\in Q}f\left(q_{i},\;D\right)$$

(2) $$f\left(q_{i},D\right)=\text{log}\left(\frac{I(tf_{q_{i},D}>0)(tf_{q_{i},D}+\delta )+\mu \frac{cf_{q_{i}}}{\left|C\right|}}{\left|D\right|+\mu }\right)$$

In Equation (1), P is a sortable quantifier of relevance. D is a dataset with metadata, Q is an input (e.g. a question), q are words in the input and $f\left(q_{i},\;D\right)$ is the weight of q in the metadata of dataset D.

In Equation (2), *t*f is the number of times word q matches the metadata of dataset D, $cf_{q_{i}}$ is the number of times word q matches the metadata of the entire collection of datasets, $\left|D\right|$ is the word number of the metadata of dataset D, C is the total word number for the collection and μ is an empirical hyper-parameter that is set to 2500. Differently from the original algorithm, we added a constant δ=5, an empirical parameter to $tf_{q_{i},D}$ if it was >0 and $I(tf_{q_{i},D}>0)$ is an indicator function. This modification puts a higher weight on the existence of a word in the metadata than on the times the word occurs.

The default version of the PSD model took as input the original Q, i.e. the free-text question. Therefore, we named this version ‘PSD-allwords’. We further developed a ‘PSD-keywords’ version that analysed only keywords extracted from Q. To identify valuable keywords from free-text questions, PSD-keywords firstly calls MetaMap (32), a biomedical named entity recognizer, to identify the UMLS concepts from Q and then uses the UMLS concept set Q' as input to PSD, with the aim of eliminating the impact of less informative words in questions. In the experiments, we used the default setting of MetaMap, collected all recognized UMLS concepts and removed duplicated concepts.

#### Snippet-based query expansion method

Hiemstra stated that in order to search a document collection, the user should first prepare a document that is similar to the needed documents (33). The idea has been widely accepted and implemented, such as in relevance feedback methods (1).

One way to measure the similarity between documents is to compare the word frequencies. The closer the word distribution of a surrogate document is to that of the user’s document, the more likely the document will be relevant to the user’s query. However, neither Elasticsearch or PSD consider the difference of word distributions in users’ questions and the dataset metadata.

Based on this idea, we used Google to find surrogate documents for users’ questions, and then transformed these documents into queries for relevant datasets. Therefore, the vocabulary and the word frequencies of each original question were replaced by an expanded vocabulary and updated word frequencies. This change potentially enriches the query contents, but also introduces noise.

The original questions were sent to Google using an in-house script, and then the top 10 retrieved text documents (not limited to datasets) were concatenated into a document that served as an input to the re-ranking algorithm. Next, the Elasticsearch retrieved datasets were re-ranked based on the concatenated documents using the PSD model.

#### Ensemble method

This method was developed based on an assumption that no single method works for all tasks. Our ensemble method averaged the reciprocal of ranks from different methods, and re-ranked datasets according to the mean of rank reciprocals. We experimented with combinations of different re-ranking algorithms, and finally chose the combination of PSD-allwords and PSD-keywords. The performance of different combinations is provided in Table 3.

### Evaluation metrics

The primary metrics in the Challenge announcement was infNDCG (34). In addition, inferred average precision (infAP) (34, 35), Normalized Discounted Cumulative Gain (NDCG)@10 (34), Precision@10(+partial) and Precision@10(-partial) were also evaluated. All these metrics are always between 0 and 1, and larger values indicate better performance.

Among the metrics, infAP, Precision@10(+partial) and Precision@10(-partial) are precision-based binary-relevance metrics: infAP is the extrapolated average precision based on incomplete judgments; Precision@10(+partial) is the precision of the top 10 results when *partially relevant* is counted as *relevant*; Precision@10(-partial) is the precision of the top 10 results when *partially relevant* is counted as *irrelevant*. Since they are binary-relevance metrics, the three metrics are not very good to distinguish between relevant and partially relevant datasets. In contrast, the DCG series metrics are designed to assign a penalty for incorrect ranking, and both infNDCG and NDCG@10 can distinguish between relevant and partially relevant documents (test data are labeled with ranks e.g. 2, 1, 0 for relevant, partially relevant and irrelevant). infNDCG considers all retrieved documents, while NDCG@10 keeps the scope to the top 10 results. infNDCG is designed to handle incomplete judgments, while NDCG@10 assumes that the judgment is complete and does not penalize for missing documents. Details of the metrics are available in Supplementary Appendix S2.

Both infNDCG and infAP were computed using a tool (http://www-nlpir.nist.gov/projects/t01v/trecvid.tools/sample_eval/sample_eval.pl, accessed on 29 January 2018) from the National Institute of Standards and Technology (NIST), NDCG@10 was evaluated using TREC_EVAL (http://trec.nist.gov/trec_eval/, accessed on 29 January 2018) from NIST, and the precision was evaluated using a script provided by the Challenge organizers.

## Results

### Implementation

The pipeline was coded in Python, Java and Perl (Scripts are available from https://github.com/w2wei/dataset_retrieval_pipeline, accessed on 29 January 2018). The metadata of datasets were indexed using Elasticsearch. Third-party libraries were also used in the implementation, including MetaMap for biomedical concept recognition.

### Computation performance

The experiments were completed on an iDASH (36) cloud virtual machine with 16 processors (Intel(R) Xeon(R) CPU E7-4870 v2) and 32 GB RAM. Indexing all datasets approximately took 3 hours. PSD-allwords and PSD-keywords each required ∼4 min to re-rank 5000 dataset candidates on 45 questions.

### Annotated questions

To facilitate the pipeline development, we manually annotated 943 datasets for the provided 30 unannotated questions (Annotation is available from https://github.com/w2wei/dataset_retrieval_pipeline, accessed on 29 January 2018). The self-made gold standard was used for optimizing configurations, tuning parameters and selecting models before we submitted our results. For details of the self-annotated questions, please refer to Supplementary Appendix S3.

### Performance in the competition

We submitted results from five methods (see Table 1), Elasticsearch, PSD-allwords, PSD-keywords, SQEM and the ensemble method. These methods were evaluated on the test set of 15 questions and all 794 992 datasets.

**Table 1:** The performance of five methods in infAP, infNDCG, NDCG@10, P@10(+partial) and P@10(−partial)

Category	Method	infAP	infNDCG	NDCG@10	P@10 (+partial)	P@10 (-partial)
No re-ranking	Elasticsearch	0.2446	0.4333	0.4228	0.5200	0.2733
Re-ranking	PSD-allwords	0.2792	**0.4980**	0.6152	**0.7600**	0.3267
	PSD-keywords	0.2391	0.4490	0.4088	0.5200	0.1667
	SQEM	**0.3309**	0.4783	**0.6504**	0.7467	**0.3600**
	Ensemble	0.2801	0.4847	0.5398	0.6800	0.2400

The indices were built on the provided metadata and the additional information. All methods used automatically generated queries. Method Elasticsearch did not use any re-ranking methods. The other four methods used re-ranking algorithms. infAP is inferred average precision, infNDCG is inferred NDCG, NDCG@10 is the NDCG score on top 10 results, P@10(+partial) is the precision of top 10 results considering ‘partially relevant’ as ‘relevant,’ P@10(-partial) is the precision of top 10 results considering ‘partially relevant’ as ‘irrelevant.’.

Among the five methods, PSD-allwords achieved the highest infNDCG and the highest P@10(+partial), and SQEM was the best method in terms of infAP, NDCG@10 and P@10(-partial).

When compared with methods from other teams in the Challenge, PSD-allwords achieved the top infNDCG score among 45 submissions from 10 teams. Our best infNDCG is ∼10% higher than the best infNDCG from the other teams. The Ensemble method and SQEM placed second and third for infNDCG in the Challenge. PSD-allwords also tied for third place for P@10 (+partial).

### Breakdown analysis

The retrieval step of the pipeline includes three key features: additional information collected from online resources, standard fields in the mapping schema and query expansion using NCBI E-utilities. To understand the contribution of each feature, we evaluated the infNDCG values of the pipeline with different settings of combinations for three features (Table 2) on the 15 test questions and the associated judgements. We found that the retrieval step achieved the highest infNDCG score when all three features were included. Removing query expansion (row 4) resulted in a larger decrease when compared to the removal of either additional fields (row 2) or standard fields (row 3). This observation indicates that the contribution from query expansion is more critical than the other two features. When looked into individual features, we noticed that additional fields alone (row 5) or standard fields (row 6) alone did not improve infNDCG when compared to using no features (row 8). Combining this observation with row 1, row 2 and row 3, we inferred that there exist interactions between the features and the interactions also help improve the infNDCG performance.

**Table 2:** Comparison of the pipeline with settings of combinations of three different features

	Additional fields	Standard fields	Query expansion	infNDCG
1	Y	Y	Y	0.4333
2	N	Y	Y	0.4164
3	Y	N	Y	0.4159
4	Y	Y	N	0.3961
5	Y	N	N	0.3868
6	N	Y	N	0.4015
7	N	N	Y	0.4084
8	N	N	N	0.4019

infNDCG measurements are scored in the rightmost column. When both additional fields and standard fields were excluded, all fields in the metadata were searched.

Y, the feature is included; N, the feature is not included.

For the ensemble method, we explored all combinations of PSD methods and SQEM (Table 3), and evaluated their performance on the 15 test questions.

**Table 3:** The performance of the Ensemble methods

PSD-allwords	PSD-keywords	SQEM	infAP	infNDCG	NDCG@10	P@10 (+partial)	P@10 (-partial)
Y	Y	Y	0.3120	0.4560	0.6089	0.7267	0.3067
N	Y	Y	0.3120	0.4442	0.5649	0.6800	0.2800
Y	N	Y	0.3216	0.4735	0.6439	0.7733	0.3333
Y	Y	N	0.2801	**0.4847**	0.5398	0.6800	0.2400

Y, the feature is included; N, the feature is not included.

## Discussion

An important aim of the Challenge was to examine if linked evidence could improve retrieval performance. In the study, we collected additional fields ‘Summary’, ‘Title’ and ‘Overall design’ for 158 963 datasets from Arrayexpress, Gemma and GEO databases to enrich the metadata of the datasets. In a breakdown analysis, we found that including additional information improved the performance of our pipeline. In the meantime, the performance may be further improved if the additional fields are refined and irrelevant information is filtered.

Another aim of the Challenge was to automatically generate queries from users’ questions. In our pipeline, we defined rules to extract keywords from questions, and to select concepts from the MetaMap output. Since these rules were pre-defined, it was inevitable that some information got lost when questions were converted into queries. Machine learning methods may provide new solutions for this problem. For example, using deep learning methods, questions may be translated into sentence embeddings to preserve all key information, and the sentence embeddings could act as queries for more effective dataset retrieval. We may pursue this approach in future work.

SQEM used the commercial search engine Google to collect relevant documents, and then identified relevant datasets using the top retrieved results. The rationale was that commercial search engines have been well optimized, and we may use their results as features in our ranking methods. We used only unigrams as features in this project. Therefore, it is possible that the performance of this re-ranking method may be further improved if better features are extracted and noise is removed. The performance of SQEM is slightly higher than PSD-allwords on the test set in terms of infAP, NDCG@10 and P@10(-partial). We compared the scores on each test question: complete results are included in Supplementary Appendix S4. Overall, the current test set is not large enough to conclude which method is actually better, and more labeled test questions will help us better understand the methods and their tradeoffs.

There are important limitations in this work. Before indexing, concepts in both the metadata and additional information were not normalized. For example, transforming growth factor beta could be written as TGF-beta, TGF beta, or TGF-β. A query containing only TGF-beta will miss datasets that only have TGF-β in the metadata. In addition, the re-ranking algorithms did not consider complicated features such as named entities, which might also help filter out ambiguous results. Finally, disambiguation methods could have been applied to the query expansion to decrease the retrieval of irrelevant datasets.

## Conclusion

We explored online resources to collect additional information and supplement the metadata of datasets, designed and implemented methods to automatically interpret users’ questions and formulate queries complying with the retrieval system, and developed a ‘retrieve plus re-rank’ strategy to identify the most relevant datasets. Our pipeline achieved the highest infNDCG score in the Challenge using a new ranking method (PSD-allwords). The Ensemble method and SQEM placed second and third in terms of infNDCG in the Challenge. The breakdown analysis suggests that the additional information and NCBI E-utilities-based query expansion also helped improve the infNDCG. In summary, we provided a promising solution for cross-database, cross-domain, cross-repository biomedical dataset retrieval.

## Supplementary data

Supplementary data are available at *Database* Online.

## Funding 

This project was supported by the National Institute of Allergy and Infectious Diseases, the National Institutes of Health grant U24AI117966.

*Conflict of interest*. None declared.

## Supplementary Material

Supplementary Data
