# Supplementary material for: Finding relevant biomedical datasets: the UC San Diego solution for the bioCADDIE Retrieval Challenge
Source: Database (Oxford). 2018 Mar 16;2018:bay017. doi: 10.1093/database/bay017 (PMC5861401; doi:10.1093/database/bay017)
Supplement: Supplementary Data [file bay017_supp.zip › Appendix_C_v2.docx]

Appendix C

We created rules to implement the relevance criteria provided by the Challenge organizers.

Figure 3 illustrates the decision process. Given a question,

1. if a dataset captures all *key concepts* in the question AND it *answers the question*, the dataset is *relevant*
2. if a dataset contains all of the key concepts but doesn’t answer the question, the dataset is *partially relevant*. For example, a concept occurred in both question and dataset has different meanings (i.e., polysemy)
3. if a dataset contains >50% of key concepts, including *components* that keeps the meaning of the associated key concepts, the dataset is partially relevant
4. if <50% of the concepts exist, including components that keep the meaning of the associated key concepts, the dataset is irrelevant

Glossary:

key concept: biomedical concepts that are included in the UMLS.

*component*: an individual word in a concept.

*keep* the meaning: a component has the same meaning as the entire concept in a context. For example, “mutation” has the same meaning as “gene mutation” in some context.

*Polysemy*: a concept has different meanings in different contexts.

*answer the question*: all curators agree that the dataset is relevant to answer the question. This is a subjective decision based on domain knowledge


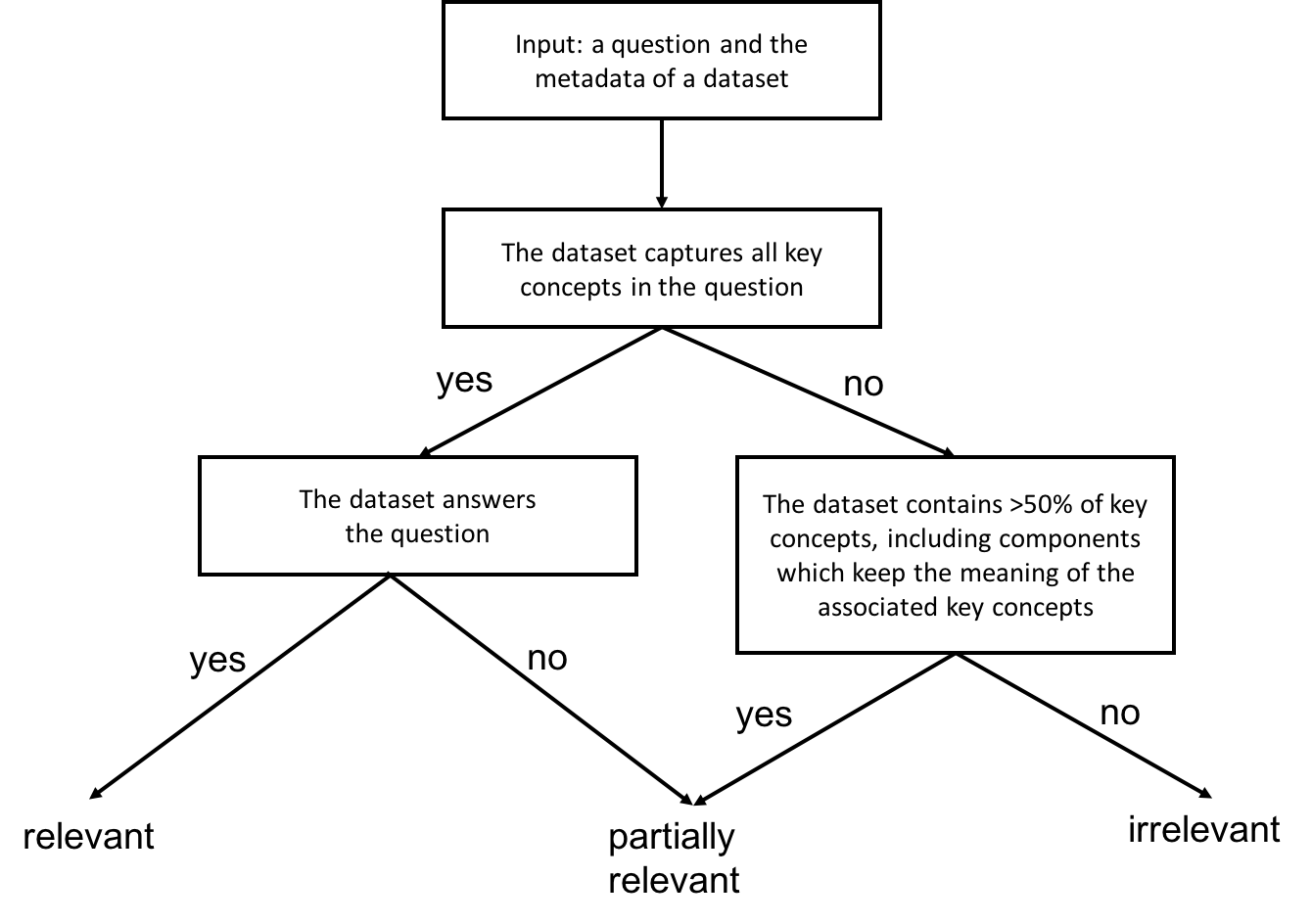


Figure 3. The decision tree for annotating questions
