# Supplementary material for: Finding relevant biomedical datasets: the UC San Diego solution for the bioCADDIE Retrieval Challenge
Source: Database (Oxford). 2018 Mar 16;2018:bay017. doi: 10.1093/database/bay017 (PMC5861401; doi:10.1093/database/bay017)
Supplement: Supplementary Data [file bay017_supp.zip › Appendix_D_v2.docx]

Appendix D

For the NDCG@10 and P@10(-), there is no significant difference between SQEM and PSD_allwords (Table 4 and 5). The difference in infAP is significant for this small test set.

The difference in infAP may be explained by the difference in query contents, i.e., the snippets returned from Google might contain additional information that is relevant to users’ needs and therefore can augment the distribution of query contents. Also, the difference may be relevant to infAP, which does not distinguish between relevant and partially relevant datasets, resulting in more relevant datasets for SQEM and higher infAP scores on this test set.

Overall, more test questions will help us better understand these methods and measure their performance by infAP, NDCG@10, and P@10(-partial)

Table 4. The infAP, NDCG@10, and P@10(-partial) on 15 test questions.

|  | infAP | | | NDCG@10 | | | P@10(-) | | |
| --- | --- | --- | --- | --- | --- | --- | --- | --- | --- |
| Question | PSD-allwords | SQEM | diff | PSD- allwords | SQEM | diff | PSD-allwords | SQEM | diff |
| 1 | 0.2946 | 0.3079 | -0.0133 | 0.5277 | 0.7403 | -0.2126 | 0.4 | 0.5 | -0.1 |
| 2 | 0.4942 | 0.5289 | -0.0347 | 0.8854 | 0.7420 | 0.1434 | 0.5 | 0.3 | 0.2 |
| 3 | 0.1558 | 0.3551 | -0.1993 | 0.553 | 0.6463 | -0.0933 | 0.2 | 0.2 | 0.0 |
| 4 | 0.5542 | 0.4698 | 0.0844 | 0.8434 | 0.5836 | 0.2598 | 0.6 | 0.5 | 0.1 |
| 5 | 0.0714 | 0.1490 | -0.0776 | 0.1811 | 0.3062 | -0.1251 | 0.0 | 0.0 | 0.0 |
| 6 | 0.2847 | 0.2173 | 0.0674 | 0.7838 | 0.6846 | 0.0992 | 0.7 | 0.4 | 0.3 |
| 7 | 0.1540 | 0.2084 | -0.0544 | 0.5834 | 0.4537 | 0.1297 | 0.0 | 0.0 | 0.0 |
| 8 | 0.0743 | 0.0422 | 0.0321 | 0.1335 | 0.4178 | -0.2843 | 0.0 | 0.2 | -0.2 |
| 9 | 0.2871 | 0.4597 | -0.1726 | 0.3839 | 0.5855 | -0.2016 | 0.1 | 0.5 | -0.4 |
| 10 | 0.2589 | 0.3258 | -0.0669 | 0.2883 | 0.1843 | 0.1040 | 0.0 | 0.0 | 0.0 |
| 11 | 0.4751 | 0.5879 | -0.1128 | 0.9526 | 1.0000 | -0.0474 | 0.9 | 1.0 | -0.1 |
| 12 | 0.4561 | 0.4957 | -0.0396 | 0.5406 | 0.5232 | 0.0174 | 0.1 | 0.1 | 0.0 |
| 13 | 0.1764 | 0.3122 | -0.1358 | 0.9052 | 1.0000 | -0.0948 | 0.9 | 1.0 | -0.1 |
| 14 | 0.3880 | 0.4295 | -0.0415 | 0.6660 | 0.8877 | -0.2217 | 0.5 | 0.7 | -0.2 |
| 15 | 0.0636 | 0.0736 | -0.0100 | 1.0000 | 1.0000 | 0.0000 | 0.0 | 0.0 | 0.0 |

Table 5. The p-values of paired two-tailed Mann-Whitney test. Based on the p values, the differences for NDCG@10 and P@10(-partial) were not significant, while the difference in infAP was significant on this test set at significance level 0.05.

|  | infAP | NDCG@10 | P@10(-) |
| --- | --- | --- | --- |
| paired two-tailed Mann-Whitney | 0.0353 | 0.5302 | 0.5497 |
