# Supplementary material for: Finding relevant biomedical datasets: the UC San Diego solution for the bioCADDIE Retrieval Challenge
Source: Database (Oxford). 2018 Mar 16;2018:bay017. doi: 10.1093/database/bay017 (PMC5861401; doi:10.1093/database/bay017)
Supplement: Supplementary Data [file bay017_supp.zip › Appendix_A_v2.docx]

Appendix A. The standard fields for 20 data repositories

YPED

"dataset.title","dataset.description", "organism.name"

ProteomeXchange

"dataset.title", "keyword","organism.name"

PhysioNet

"dataset.title","dataset.description","organism.name"

Phenodisco

"topic", "MESHterm", "phenCUI"(UMLS concepts), "title", "Demographics", "demographics","inexclude", "desc","disease", "phenDesc", "gender", "organism.name"

Peptideatlas

"dataset.title","dataset.description","treatment.description", "organism.name","organism.strain"

PDB

"materialEntity.name", "dataItem.keywords", "dataItem.title", "dataItem.description","citation.title", "gene.name", "organism.source.scientificName", "organism.source.strain", "organism.host.scientificName", "organism.host.strain"

Openfmri

"dataset.title","dataset.description", "organism.name"

Nursadatasets

"publication.description", "dataset.keywords", "dataset.title","dataset.description", "organism.name"

Neuromorpho

"dataset.title", "dataset.note", "treatment.title", "organism.strain","organism.scientificName","organism.name", "organism.gender", "anatomicalPart.name"

MPD

"dataset.title","dataset.description", "organism.strain","organism.scientificName","organism.name", "dataset.gender"

GEO

"dataItem.description","dataItem.title", "organism", "source_name"

GEMMA

"dataItem.title","dataItem.description", "organism.source.commonName"

Dryad Data Repository

"dataset.title", "dataset.keywords"

Dataverse Network Project

"publication.description", "dataset.title","dataset.description"

CVRG

"dataset.title","dataset.description"

CTN

"dataset.title","dataset.description", "organism.scientificName", "organism.name"

Clinicaltrials

"Study.recruits.criteria" (inclusion and exclusion criteria), "Treatment.description", "Dataset.briefTitle", "Dataset.keyword", "Dataset.title","Dataset.description"

CIA

"disease.name", "dataset.title", "anatomicalPart.name", "organism.scientificName", "organism.name"

Bioproject

"dataItem.description","dataItem.title","dataItem.keywords", "organism.target.species"

Arrayexpress

"dataItem.description","dataItem.title"
