# Supplementary material for: Finding relevant biomedical datasets: the UC San Diego solution for the bioCADDIE Retrieval Challenge
Source: Database (Oxford). 2018 Mar 16;2018:bay017. doi: 10.1093/database/bay017 (PMC5861401; doi:10.1093/database/bay017)
Supplement: Supplementary Data [file bay017_supp.zip › Appendix_B_v2.docx]

Appendix B. Evaluation metrics

The evaluation metrics of the Challenge include precision@10, inferred average precision (infAP) (1), Normalized Discounted Cumulative Gain (NDCG)@10 (2), and inferred Normalized Discounted Cumulative Gain (infNDCG) (2).

### Precision

Precision ($P$) measures the percentage of relevant documents in all the retrieved documents,

$$P=\frac{number of relevant documents}{number of retrieved documents}$$

and Precision@10 ($P@10$) is

$$P@10=\frac{number of relevant documents in top 10 results}{10}$$

### Average precision (AP) and inferred average precision (infAP)

AP is a widely used system-oriented measure in information retrieval research. AP is the average of precision at each relevant document,

$$AP=\frac{\sum_{i=1}^{n} (P(i)\times rel(i))}{number of relevant documents}$$

where $P(i)$ is the precision at the $i^{th}$ document, and $rel(i)$ is the relevance of the $i^{th}$ document (0 for irrelevant, 1 for relevant), $n$ is the number of retrieved documents.

When the relevance judgments are incomplete, $P(i)$ is estimated using sampling methods (1). Therefore,

$$infAP=\frac{\sum_{k=1}^{n} (\hat{P(i)}\times rel(i))}{number of relevant documents}$$

where $\hat{P(i)}$is the expected precision at the $i^{th}$ document. More information is available in Yilmaz & Aslam’s work (1).

### NDCG, NDCG@10 and infNDCG

Normalized Discounted Cumulative Gain (NDCG) (3) is based on Discounted Cumulative Gain (DCG) (3). DCG is a weighted sum of the relevance of the ranked documents, and the weight is a decreasing function of the rank of the document (4). The definition of DCG is,

$$DCG_{n}=\sum_{i=1}^{n} \frac{rel_{i}}{\log_{2} (i+const)}$$

where $n$ is the number of retrieved documents, $rel_{i}$ is the graded relevance (0 for irrelevant, 1 for partially relevant and 2 for relevant) of the $i^{th}$ document, and $const$ is a smoothing constant. In TREC_EVAL 9.0, $const=2$.

NDCG normalizes DCG by the ideal DCG, which is the DCG of the best ranking list (i.e. retrieved documents ordered by the relevance values) (4),

$$NDCG_{n}=\frac{DCG_{n}}{IDCG_{n}}$$

$$IDCG_{n}=\sum_{i=1}^{\left| REL \right|} \frac{rel_{i}}{\log_{2} \left( i+const \right)}$$

where $REL$ is the best ranking list, and $|REL|$ is the size of the best ranking list.

When only the top $i$ documents are considered, NDCG is referred to as NDCG@i (4). Therefore,

$$NDCG@10=\frac{DCG_{10}}{IDCG_{10}}$$

When the relevance judgments are incomplete, $DCG_{n}$ and $IDCG_{n}$ are estimated using sampling methods (2),

$$infNDCG_{n}=\frac{\hat{DCG_{n}}}{\hat{IDCG_{n}}}$$

where $\hat{DCG_{n}}$ is the expected $DCG_{n}$, and $\hat{IDCG_{n}}$ is the expected $IDCG_{n}$.

# References

1. Yilmaz E, Aslam JA. Inferred AP : estimating average precision with incomplete judgments. In: Fifteenth ACM International Conference on Information and Knowledge Management. Arlington, Virginia, USA: ACM Press; 2006. p. 102–11.

2. Yilmaz E, Kanoulas E, Aslam JA. A simple and efficient sampling method for estimating AP and NDCG. In: Proceedings of the 31st Annual International ACM SIGIR Conference on Research and Development in Information Retrieval. Singapore, Singapore; 2008. p. 603–10.

3. Järvelin K, Kekäläinen J. Cumulated gain-based evaluation of IR techniques. ACM Trans Inf Syst. ACM; 2002 Oct 1;20(4):422–46.

4. Wang Y, Wang L, Li Y, He D, Liu T-Y. A theoretical analysis of NDCG type ranking measures. In: Proceedings of the 26th Annual Conference on Learning Theory. Princeton, NJ, USA: JMLR; 2013. p. 25–54.
